# Lightweight deep learning for medical imaging using MobileNetV2-based brain pathology classification with Grad-CAM interpretability

**DOI:** 10.3389/fmed.2026.1810860

**Published:** 2026-05-29

**Authors:** Mian Usman Sattar, Meznah A. Alamro, Alaeddine Mihoub, Soliman Aljarboa, Moez Krichen

**Affiliations:** 1 Department of Computing, University of Derby, Derby, United Kingdom; 2 Department of Information Technology, College of Computer and Information Sciences, Princess Nourah bint Abdulrahman University, Riyadh, Saudi Arabia; 3 Department of Management Information Systems, College of Business and Economics, Qassim University, Saudi Arabia; 4 Faculty of Computing and Information, Al-Baha University, Al-Baha, Saudi Arabia; 5 Redcad Laboratory, University of Sfax, Sfax, Tunisia

**Keywords:** convolutional neural networks, CT brain scans, ensemble prediction, medical image classification, transfer learning

## Abstract

**Introduction:**

Computed Tomography (CT) brain scans are crucial for diagnosing various neurological conditions, including tumors, cancer, and aneurysms. CT brain scans are essential for guiding treatment decisions and monitoring disease progression. In this study, we propose a novel framework for brain CT image classification that leverages convolutional neural networks (CNNs), Digital Imaging and Communications in Medicine (DICOM) pre-processing, transfer learning with multiple deep models, and ensemble prediction techniques. The primary objective is to enhance the accuracy and interpretability of brain abnormality detection.

**Methods:**

Our approach uses a comprehensive dataset of CT brain scans that undergo meticulous pre-processing to ensure data integrity and uniformity, and employs Grad-CAM for interpretability. We employ four state-of-the-art pre-trained models: MobileNetV2, ResNet-50, EfficientNet-B0, and VGG-16, each serving as a feature extractor, followed by a classification head tailored to our specific task.

**Results:**

The experimental results demonstrate that MobileNetV2 and the Ensemble model achieved the highest classification accuracy of 97.44% with macro-AUC scores of 0.9895 and 0.9914, respectively, followed by VGG16 with 92.31% accuracy and the highest macro-AUC of 0.9962. In contrast, ResNet50 and EfficientNetB0 achieved accuracies of 61.54% and 33.33%, respectively, indicating fundamental limitations in learning discriminative features for this medical imaging task. MobileNetV2 proved to be the most efficient model, achieving superior accuracy with training and test times of 89 s and 38.16 s, respectively.

**Discussion:**

MobileNetV2 is highly suitable for clinical deployment where both accuracy and computational efficiency are critical.

## 1 Introduction

The human brain is the most intricate and vital organ in the body, responsible for regulating nearly all bodily functions, ranging from voluntary movements and decision-making to memory and emotional response (1). Given its central role, any abnormality affecting the brain can have deep and often life-threatening consequences (2). Among the most serious conditions affecting the brain are tumors, aneurysms, and brain cancer. These disorders vary significantly in origin and progression but share the common challenge of requiring timely and accurate diagnosis (3). Brain tumors are abnormal masses of tissue resulting from uncontrolled cell growth. They may be benign, with slow growth and clear boundaries, or malignant, showing aggressive infiltration and rapid progression (4). Brain cancer refers explicitly to malignant tumors and often carries a poor prognosis due to its fast spread and resistance to treatment (5). Cerebral aneurysms, on the other hand, involve the abnormal bulging or ballooning of blood vessels in the brain. If ruptured, aneurysms can cause life-threatening hemorrhages and require urgent medical attention (6). Early detection of all three conditions is crucial; however, clinical diagnosis remains challenging due to overlapping symptoms, limited resources, and heavy reliance on expert interpretation.

In this context, Computed Tomography (CT) of the brain has emerged as a frontline imaging modality, particularly in emergency and acute care settings (7). CT imaging utilizes X-ray beams and computer processing to generate detailed cross-sectional images of the brain. It is preferred in scenarios that require rapid visualization of brain structures, hemorrhages, masses, or abnormal vascular conditions, such as aneurysms (8). Since its clinical adoption in the 1970s, CT brain imaging has undergone tremendous technological advancements, enabling faster acquisition times, higher resolution, and reduced radiation exposure. CT is particularly valuable in regions with limited access to MRI scanners, owing to its wider availability, speed, and diagnostic utility for both trauma and pathology (9). Despite these advantages, manual interpretation of CT brain scans is a complex and time-consuming task that heavily depends on radiologists' expertise (10). The increasing volume of imaging data and the presence of subtle pathological signs pose significant challenges for accurate and consistent diagnoses (11). This has paved the way for the integration of Artificial Intelligence (AI), particularly Deep Learning (DL), into medical imaging workflows (12).

In this study, we present a robust AI-driven framework for the automated classification of CT brain images using deep convolutional neural networks (CNNs). The dataset employed includes a diverse set of Digital Imaging and Communications in Medicine (DICOM)-formatted CT brain scans labeled for critical conditions such as tumors, aneurysms, and brain cancer. The proposed framework aims not only to improve diagnostic accuracy but also to provide transparent and explainable results that support clinical decision-making. By leveraging AI in CT imaging, our work contributes to the development of scalable, practical tools for early, reliable diagnosis of brain diseases.

The three major contributions of this study are the following:

- Designed a robust framework for brain CT image classification that integrates DICOM pre-processing, transfer learning, and ensemble prediction to address challenges in medical image analysis and enhance classification accuracy.
- The proposed approach employs four state-of-the-art pre-trained CNN architectures (MobileNetV2, ResNet50, EfficientNetB0, and VGG16) as feature extractors, demonstrating the effectiveness of transfer learning for extracting relevant features from medical images.
- Demonstrated that MobileNetV2 and the Ensemble model achieved the highest classification accuracy of 97.44% with macro-AUC scores of 0.9895 and 0.9914, respectively, followed by VGG16 with 92.31% accuracy and the highest macro-AUC of 0.9962. In contrast, ResNet50 and EfficientNetB0 achieved accuracies of 61.54% and 33.33%, respectively, indicating fundamental limitations in learning discriminative features for this medical imaging task. MobileNetV2 proved to be the most efficient model, achieving superior accuracy with training and test times of 89 s and 38.16 s, respectively, making it highly suitable for clinical deployment where both accuracy and computational efficiency are critical.

The remainder of this paper is organized as follows: Section 2 provides a comprehensive review of related work focusing on brain disease classification using medical imaging and deep learning techniques. Section 3 presents the dataset description and the proposed methodology, which includes DICOM pre-processing, deep convolutional neural networks, transfer learning, ensemble classification strategies, and the GRAD CAM method for interpretability. Section 4 discusses the experimental setup, evaluation metrics, and performance analysis of individual and ensemble models. Finally, Section 5 concludes the paper and outlines future directions for enhancing diagnostic accuracy and real-time deployment in clinical settings.

## 2 Related work

### 2.1 Deep learning for brain CT diagnosis and classification

Deep learning, a branch of machine learning based on multi-layer artificial neural networks, learns hierarchical feature representations directly from imaging data (13). This approach—especially convolutional neural networks (CNNs)—has transformed medical image analysis by enabling automated detection, segmentation, and classification of subtle and complex patterns on CT scans with far less manual feature engineering than traditional methods. CNNs have been widely applied to head CT for automated diagnosis of acute brain conditions, including intracranial hemorrhage (ICH), stroke, brain tumor, and metastases. CNN-based systems on non-contrast CT have achieved high performance in stroke detection and binary normal/abnormal classification, with accuracies around 97% and external test performance near 90% on independent cohorts (14). For ICH, several works classify multiple hemorrhage subtypes (epidural, intraventricular, subarachnoid, intraparenchymal, subdural) from CT, reaching per-class accuracies above 98% and overall F1-Score around 0.96–0.97 (15–18). Radiomics-based machine learning ensembles have also been used to distinguish brain metastases, brainstem, and normal tissue, where stacking ensembles attain AUCs ≈0.93-0.94 and outperform single classifiers across multiple evaluation metrics (19).

Most existing CT studies focus on a single or narrow clinical indication, such as Intracerebral hemorrhage (ICH) or stroke, and many treat the problem as binary detection rather than multi-class abnormality categorization. Brain tumors and aneurysms are relatively underrepresented compared to hemorrhage and trauma. Additionally, model evaluation is often task-specific (e.g., ICH vs. non-ICH), limiting direct generalization to broader multi-class brain abnormality triage scenarios (14, 15, 19).

### 2.2 Transfer learning with pre-trained CNNs for brain imaging

Transfer learning leverages neural networks pre-trained on large natural-image datasets to provide rich, general-purpose feature representations that can be fine-tuned for medical imaging tasks (20). By initializing models with learned low- and mid-level filters (edges, textures, shapes), transfer learning reduces the need for large labeled medical datasets, speeds convergence, and often improves accuracy and robustness when applied to CT or MRI through careful augmentation and domain-specific fine-tuning.

Transfer learning from large-scale natural image datasets is now standard in medical image analysis, given the limited availability of labeled CT/MRI data. In CT brain tumor detection, a correlation-learning mechanism that augments a CNN with a support network achieved ≈96% accuracy and ≈95% precision/recall, highlighting the benefit of more expressive deep representations in CT despite modest dataset sizes (21). Other CT ICH and fusion models use modern pre-trained backbones (e.g., DenseNet-121) as feature extractors before temporal modeling or decision layers, achieving accuracies of 97-99% on multi-class hemorrhage tasks (15, 16). Although much of the detailed benchmarking of ImageNet-pre-trained architectures has been performed on MRI, these results inform architecture choices for CT. Studies comparing VGG-16, ResNet-50, Inception-v3, MobileNetV2, DenseNet, and EfficientNet on brain tumor MRI consistently report strong performance for transfer learning, even on small datasets, with accuracies often above 98-99% under adequate augmentation and fine-tuning (22–25). Survey and review articles further emphasize the advantages of CNN-based transfer learning in medical imaging, particularly improved accuracy, reduced training time, and robustness on limited data (26, 27).

However, CT-specific analyses of transfer learning remain relatively sparse compared with MRI, and there is limited systematic comparison of multiple pre-trained backbones (e.g., MobileNetV2, ResNet-50, EfficientNet-B0, VGG-16) within a single framework for brain CT abnormalities. Existing CT methods often rely on a single architecture or fail to exploit complementary feature representations across multiple CNNs (15, 16, 21).

### 2.3 Ensemble approaches for robust medical image classification

Ensemble learning combines multiple models to reduce variance, mitigate individual model biases, and improve overall predictive performance (28). By aggregating complementary classifiers or feature extractors—via bagging, boosting, stacking, or simple averaging—ensembles increase robustness to dataset shifts and often yield higher accuracy, calibration, and reliability than single models in medical image classification. Ensemble learning is increasingly recognized as a way to improve robustness and generalization in medical image classification. A broad analysis of ensemble strategies across several medical imaging datasets showed that stacking and cross-validation-based bagging can improve F1-Score by up to 11–13%. At the same time, simple averaging often performs as well as more complex pooling functions (29).

In the CT domain, ensemble techniques have been employed at both the feature and prediction levels. For ICH classification from CT, an ensemble of SE-ResNeXt and LSTM networks, combined at the decision level, achieved near-perfect accuracy (≈99.8%) and high AUC across five hemorrhage subtypes (15). Voting and meta-ensemble schemes that integrate multiple deep learners (e.g., RNN, BiLSTM, stacked autoencoders) have also been proposed for ICH diagnosis, improving performance over individual models (17). Radiomics-based stacking ensembles similarly outperform single algorithms for CT classification of brain metastases (19).

Despite these advances, most CT ensembles are designed for specific pathologies (e.g., ICH) and do not systematically combine heterogeneous pre-trained CNN backbones as complementary feature extractors. Furthermore, few studies explicitly investigate the benefits of ensembles in multi-class, multi-abnormality settings that include tumor or aneurysm classes.

### 2.4 DICOM-aware pre-processing, CT windowing, and HU normalization

DICOM-aware pre-processing converts raw CT data into standardized, analysis-ready images by applying Hounsfield Unit (HU) calibration, clinically informed windowing, artifact removal, and spatial normalization. These steps preserve quantitative CT information and harmonize inputs across scanners and protocols, enabling models to learn clinically meaningful tissue contrasts (e.g., blood vs. parenchyma) rather than scanner-specific intensity patterns. Reliable learning from CT requires appropriate handling of raw DICOM data, including Hounsfield Unit conversion, windowing, and artifact removal. Methodological work on head CT processing recommends an end-to-end pipeline from raw DICOM to standardized brain volumes, including anonymization, DICOM-to-NIfTI conversion, skull stripping, and registration to a CT template; these steps are crucial for harmonizing data from heterogeneous scanners and protocols (30). For clinical hemorrhage tasks, pre-processing typically includes windowing to brain, subdural, and bone windows, with three-window stacking used to enhance contrast between soft tissues and blood (15). More advanced approaches explicitly model HU-to-RGB transformations with automatic window-width and window-level selection to emphasize hemorrhagic regions, significantly improving sensitivity and specificity for five-way ICH subtype classification compared with direct reading of DICOM images (31). Other CT-based diagnostic models show that carefully standardizing the dynamic range for brain structures and removing high-intensity artifacts (e.g., scanner bed) can materially change CNN attention patterns and improve downstream performance (32). However, many deep learning studies treat CT images as generic grayscale inputs, with limited discussion of DICOM header handling, HU normalization, or clinically motivated window selection. Systematic DICOM-aware pre-processing tailored for multi-class brain abnormality classification (including tumor and aneurysm) remains underexplored.

### 2.5 Interpretability for brain CT CNNs

Interpretability—defined as methods that make a model's decision process understandable to humans by identifying the inputs, regions, or features that drive predictions—is essential for validating that CNNs for brain CT attend to clinically meaningful anatomy and for detecting failure modes (33). Model interpretability is essential for clinical adoption of deep learning in neuroimaging. For CT stroke detection, explanation techniques such as Local Interpretable Model-Agnostic Explanations (LIME), occlusion sensitivity, and saliency maps have been applied to visualize which regions drive CNN predictions, revealing clinically plausible focus on ischemic or hemorrhagic areas and supporting model transparency (14). In multi-class ICH CT classification, Grad-CAM is commonly used to highlight the region of interest and verify that the ensemble model attends to hemorrhage rather than artifacts (15). More specialized CT-based work on mild traumatic brain injury uses Grad-CAM and occlusion sensitivity maps within an interpretable 3D multimodal residual network, showing that noise removal and appropriate pre-processing shift attention from scanner artifacts to brain parenchyma, and that occlusion-based maps provide sharper localization than Grad-CAM (32). In MRI-based tumor diagnosis, occlusion sensitivity and other explainable AI (XAI) techniques (Grad-CAM++, LIME, SHAP) have been used to generate heatmaps that align with radiological expectations, and some studies report that occlusion analysis may yield more spatially precise explanations than gradient-based methods (34, 35).

Nonetheless, interpretability in CT brain classification is often limited to a single technique (usually Grad-CAM) and is rarely integrated as a systematic, quantitative component of model design and evaluation. Comparative analyses of occlusion-based vs. gradient-based interpretability for multi-class CT brain abnormalities remain limited (14, 32).

### 2.6 Positioning of the present work

Existing research demonstrates strong performance of CNN-based models for specific CT brain tasks (primarily ICH and stroke), the value of transfer learning from ImageNet-pre-trained architectures, the benefits of ensemble learning, and the importance of CT-specific pre-processing and interpretability. However, (see Table 1) prior work typically addresses a single pathology, uses one backbone architecture, gives limited attention to DICOM-aware pre-processing, or restricts interpretability to basic Grad-CAM visualizations (14, 15, 19, 29–32).

**Table 1:** Summary of methods and findings for brain CT/MRI deep-learning components.

Topic	Key points	Gaps/limitations
Deep learning for diagnosis	CNNs learn hierarchical features from imaging; applied to ICH, stroke, tumors, metastases. High reported performance: stroke binary classification ≈97% accuracy (external test ≈90%); multi-class ICH per-class accuracies >98%, F1 ≈0.96–0.97; radiomics ensembles AUC ≈0.93–0.94.	Focus often on single indications (ICH, stroke); many studies treat problems as binary; tumors and aneurysms are underrepresented; limited generalization to multi-class triage.
Transfer learning with pre-trained CNNs	Pre-training on large natural-image datasets provides reusable low- and mid-level filters that speed convergence and improve accuracy on small medical datasets. CT/MRI studies use backbones (DenseNet, ResNet, VGG, MobileNetV2, EfficientNet) with reported accuracies often >96–99% after fine-tuning.	CT-specific systematic comparisons of multiple backbones are sparse; many CT works use a single architecture and do not exploit complementary representations across models.
Ensemble approaches	Aggregating models (bagging, stacking, averaging) reduce variance and bias; stacking/CV-bagging can improve F1 by 11–13%; simple averaging is often competitive. CT ensembles (feature- or decision-level) show strong results (e.g., SE-ResNeXt + LSTM ≈99.8% accuracy for ICH).	Most ensembles target specific pathologies (ICH); few studies systematically combine heterogeneous pre-trained CNN backbones; evaluation is limited in multi-class, multi-abnormality settings.
DICOM-aware pre-processing, windowing, HU normalization	Convert to Hounsfield Units, apply clinically informed windowing (brain/subdural/bone), artifact removal, skull stripping, registration; three-window stacking and HU → RGB/window selection improve contrast for hemorrhage and model sensitivity/specificity. Standardization changes CNN attention and downstream performance.	Many studies treat CT as a generic grayscale without DICOM header handling or HU normalization; systematic DICOM-aware pre-processing for multi-class brain abnormality classification is underexplored.
Interpretability for brain CT CNNs	Interpretability methods (saliency, occlusion sensitivity, Grad-CAM, SHAP/LIME) visualize regions driving predictions; applied in stroke, ICH, TBI, and MRI tumor studies. Occlusion often yields sharper localization; gradient-based methods are widely used (e.g., Grad-CAM).	Interpretability is often limited to a single technique (typically Grad-CAM); rarely quantitative or integral to evaluation; few comparative analyses of occlusion- vs. gradient-based methods for multi-class CT abnormalities.

Unlike these approaches, the proposed study targets multi-class classification of CT brain abnormalities (including tumors, malignancies, and aneurysms). It integrates (i) explicit DICOM-based pre-processing with clinically motivated windowing and normalization, (ii) multiple pre-trained CNN feature extractors (e.g., MobileNetV2, ResNet-50, EfficientNet-B0, VGG-16), (iii) ensemble prediction to enhance robustness across classes, and (iv) Grad Cam interpretability complemented by saliency-style methods. This combination directly addresses current gaps in generalization, DICOM awareness, and explainability in the comprehensive triage of brain abnormalities on CT.

## 3 Proposed framework

**Figure 1:**
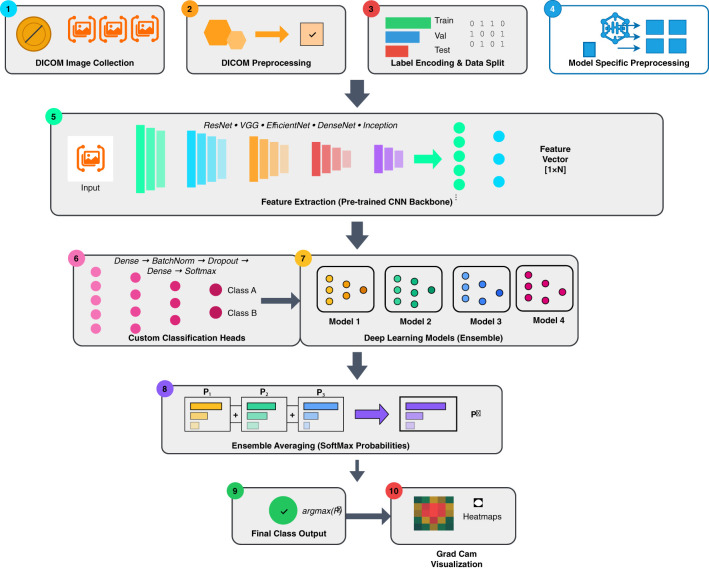
Proposed framework for brain CT image classification.

This section presents the detailed design and mathematical formulation of our proposed framework for brain CT image classification, which employs transfer learning, DICOM pre-processing, multi-model transfer learning, and ensemble prediction. The entire pipeline emphasizes both classification accuracy and interpretability through Grad Cam. The proposed framework for brain CT image classification is represented in Figure 1.

### 3.1 Experimental dataset preparation and pre-processing

The dataset ^1^ consists of CT brain scan images representing three critical neurological conditions: tumors, cancers, and aneurysms. Each scan provides a detailed view of the patient's brain using Computed Tomography (CT), enabling accurate medical image analysis. The data are available in two formats: JPG and DICOM (.dcm). The DICOM format preserves detailed metadata and image quality for clinical use. The tumor category encompasses abnormal tissue growth that can be benign or malignant. Aneurysms are characterized by bulging blood vessels due to weakened vessel walls, which, if ruptured in the brain, can result in life-threatening strokes or hemorrhages. The cancer category refers to malignant brain growths, such as gliomas, known for aggressive and uncontrolled cell proliferation. This diverse dataset supports the development and validation of deep learning models for the accurate classification of brain abnormalities. The classification pipeline begins by reading and preparing CT scan images, which are initially stored in the DICOM format, the standard for medical imaging.

Table 2 presents the comprehensive distribution of the medical image dataset across training, validation, and test partitions. The dataset contains 259 brain MRI images with standardized dimensions of 22 × 224 × 3 pixels, representing three pathological conditions: aneurysm (84 images, 32.43%), cancer (91 images, 35.14%), and tumor (84 images, 32.43%). Each class was split proportionally to maintain consistent ratios across subsets, with approximately 72% allocated to training, 13% to validation, and 15% to testing. This stratified partitioning strategy ensures that all three classes are adequately represented in each subset, preventing class imbalance during model training and providing reliable performance estimates during validation and testing phases. The nearly equal distribution across classes reduces potential bias and ensures that the trained models learn discriminative features across all diagnostic categories.

**Table 2:** Dataset distribution across training, validation, and test sets.

Class	Training		Validation		Test		Total	
	Count	%	Count	%	Count	%	Count	%
Aneurysm	60	71.43	11	13.10	13	15.48	84	32.43
Cancer	66	72.53	11	12.09	14	15.38	91	35.14
Tumor	61	72.62	11	13.10	12	14.29	84	32.43
Total	187	72.20	33	12.74	39	15.06	259	100

#### 3.1.1 DICOM image loading and pre-processing

Each image is first extracted from its raw DICOM file. The grayscale image is resized to a fixed 224 × 224 pixel resolution to match the input requirements of modern deep learning architectures. After resizing, pixel values are normalized to a standard range, ensuring uniformity across the dataset and facilitating stable model training. To ensure data integrity, the total number of available DICOM images and their corresponding label entries is checked. Only valid and complete entries are considered for further processing. The image-label pairs are organized by mapping textual class labels to numerical indices. Each entry in the dataset is iterated over: the corresponding image is loaded, pre-processed, and paired with its label. These pairs are collected into separate image and label arrays for later use in model training and evaluation.

Stratified Dataset Splitting: Once all images have been processed and labeled, the dataset is split into training and testing subsets. A stratified approach is used for this partitioning, ensuring that the class distribution remains consistent across both subsets. This technique helps maintain a balanced class representation and improves the reliability of the evaluation metrics. After splitting, each image is further processed to conform to the input specifications of the respective deep learning backbones used in the ensemble model. Since pre-trained models are typically trained on color images with three channels, grayscale CT images are converted to a pseudo-RGB format by duplicating the single channel across all three channels. This transformation enables compatibility with the pre-trained models without altering the core information content of the medical scans.

### 3.2 Transfer learning-based model construction

Each pre-trained model *M*_*i*_ comprises two main components: a frozen base network that extracts high-level features from the input image, and a trainable classification head that produces the final prediction based on these features. The feature extraction process is mathematically defined in Equation 1:


$$\begin{array}{c} F_{i}:{\mathbb{R}}^{h\times w\times c}\to {\mathbb{R}}^{d_{i}},\;f_{i}=F_{i}\left(X_{i}^{\text{processed}}\right) \end{array}$$ (1)

Where:

- *F*_*i*_ represents the frozen convolutional base of a pre-trained CNN (such as MobileNetV2, ResNet50, EfficientNetB0, or VGG16).
- $X_{i}^{\text{processed}}$ is the input image pre-processed according to the respective model's pre-processing function.
- $f_{i}\in {\mathbb{R}}^{d_{i}}$ is the output feature vector, capturing essential spatial and semantic information.

The pre-trained weights are preserved (frozen) to retain valuable representations learned from large-scale datasets, such as ImageNet, thereby reducing overfitting and accelerating convergence. The classification head *H*_*i*_ processes the feature vector through a series of fully connected layers activated by ReLU functions and outputs class probabilities via a softmax layer and represented in Equation 2.


(2) $$\begin{array}{l} H_{i}(f_{i})=\text{ softmax}(W_{i}^{\left(L_{i}\right)}\phi (\cdots \phi (W_{i}^{\left(1\right)}f_{i}+b_{i}^{\left(1\right)})\\ \;\cdots +b_{i}^{\left(L_{i}-1\right)})+b_{i}^{\left(L_{i}\right)}) \end{array}$$

Where:

- $W_{i}^{\left(l\right)}$ and $b_{i}^{\left(l\right)}$ denote the weights and biases of the *l*-th dense layer.
- ϕ(*x*) = max(0, *x*) is the ReLU activation function.
- *L*_*i*_ is the total number of layers in the classification head of model *M*_*i*_.
- The final softmax function produces a probability distribution over *C* target classes.

The complete forward propagation of model *M*_*i*_ for an input image *X* is given by Equation 3.


$$\begin{array}{c} M_{i}\left(X\right)=H_{i}\left(F_{i}\left(P_{i}\left(X\right)\right)\right) \end{array}$$ (3)

Where *P*_*i*_ is the pre-processing function specific to model *M*_*i*_ (e.g., resizing, normalization). Each model is trained independently using the training dataset. The objective is to minimize the categorical cross-entropy loss function, which measures the divergence between the true class distribution and the predicted one by Equation 4


$$\begin{array}{c} L\left(y,{ŷ}_{i}\right)=-\overset{C}{\underset{c=1}{\sum }}y_{c}\log\left({ŷ}_{i,c}\right) \end{array}$$ (4)

Where:

- *y*∈{0, 1}^*C*^ is the one-hot encoded true label vector.
- ŷ_*i*_ = *M*_*i*_(*X*) is the predicted class probability distribution from model *M*_*i*_.

To ensure effective training:

- The Adam optimizer is used for adaptive learning rate adjustment.
- Early stopping halts training once validation performance no longer improves.
- Learning rate reduction on a plateau slows the learning rate for finer convergence.
- Model checkpointing stores the best model based on validation accuracy.

### 3.3 Ensemble model for robust prediction

To improve generalization and reduce the variance associated with individual models, an ensemble approach is employed. This combines predictions from all base models. The ensemble model aggregates the class probabilities predicted by each of the *N* base models via averaging and is represented by Equation 5


$$\begin{array}{c} M_{\text{ensemble}}\left(X\right)=\frac{1}{N}\overset{N}{\underset{i=1}{\sum }}M_{i}\left(X\right) \end{array}$$ (5)

Where *N* = 4 corresponds to the number of models: MobileNetV2, ResNet50, EfficientNetB0, and VGG16. The final predicted class label is determined by Equation 6, selecting the class with the highest average probability:


$$\begin{array}{c} {ŷ}_{\text{ensemble}}=\arg\underset{c}{\max}{\left[M_{\text{ensemble}}\left(X\right)\right]}_{c} \end{array}$$ (6)

This ensemble method enhances classification reliability by leveraging complementary strengths of all participating deep learning models.

### 3.4 Deep learning models architecture

This section explains the architecture and functionality of the individual deep learning models used in this study, namely MobileNetV2, ResNet-50, EfficientNet-B0, and VGG-16, as well as their ensemble integration.

#### 3.4.1 MobileNetV2 transfer learning model

MobileNetV2 (36) is a lightweight convolutional neural network architecture designed for mobile and embedded vision applications. The architecture employs inverted residual blocks with linear bottlenecks to reduce computational complexity while maintaining high accuracy. In this transfer learning implementation, we utilize a pre-trained MobileNetV2 base model with ImageNet weights, freeze its layers to preserve learned features, and add a custom classification head consisting of global average pooling, a dense layer with 512 units, batch normalization for stable training, dropout regularization at 0.5 to prevent overfitting, and a final softmax output layer for multi-class classification. The full model construction is summarized in Algorithm 1.

Algorithm 1 Pseudocode of MobileNetV2 transfer learning model.

 Require:  input_shape, num_classes
 1:  inputs ← Input(input_shape)
 2:  base ← MobileNetV2(weights='imagenet', include_top=False, input_tensor=inputs)
 3:  base.trainable ← False
 4:  x ← GlobalAveragePooling2D()(base.output)
 5:  x ← Dense(512, activation='relu')(x)
 6:  x ← BatchNormalization()(x)
 7:  x ← Dropout(0.5)(x)
 8:  outputs ← Dense(num_classes, activation='softmax')(x)
 9:  return Model(inputs, outputs, name='MobileNetV2')

#### 3.4.2 ResNet50 transfer learning model

ResNet50 (37) introduces the concept of residual learning through skip connections that allow gradients to flow directly through the network, enabling the training of very deep architectures without degradation problems. The architecture comprises 50 layers organized into residual blocks, in which each block learns residual mappings rather than direct ones. Our transfer learning approach loads the pre-trained ResNet50 base without the original classification top, freezes all convolutional layers, and constructs a deeper classification head with two dense layers of 1,024 and 512 units, respectively, each followed by batch normalization and dropout layers with rates of 0.5 and 0.3 to ensure robust feature extraction and prevent overfitting on the target dataset. A pseudocode of the ResNet50 transfer learning model is shown in Algorithm 2.

Algorithm 2 Pseudocode of ResNet50 transfer learning model.

 Require:  input_shape, num_classes
 1:  inputs ← Input(input_shape)
 2:  base ← ResNet50(weights='imagenet', include_top=False, input_tensor=inputs)
 3:  base.trainable ← False
 4:  x ← GlobalAveragePooling2D()(base.output)
 5:  x ← Dense(1024, activation='relu')(x)
 6:  x ← BatchNormalization()(x)
 7:  x ← Dropout(0.5)(x)
 8:  x ← Dense(512, activation='relu')(x)
 9:  x ← BatchNormalization()(x)
 10:  x ← Dropout(0.3)(x)
 11:  outputs ← Dense(num_classes, activation='softmax')(x)
 12:  return Model(inputs, outputs, name='ResNet50')

#### 3.4.3 EfficientNetB0 transfer learning model

EfficientNetB0 (38) represents a family of models developed through compound scaling, which uniformly scales network depth, width, and resolution using a compound coefficient. This architecture achieves state-of-the-art accuracy with significantly fewer parameters than previous models by optimizing the trade-off between model size and performance. The transfer learning implementation uses pre-trained EfficientNetB0 weights, freezes the base model, and adds a custom head with two dense layers of 768 and 384 units, incorporating batch normalization after each dense layer and applying dropout at rates of 0.4 and 0.3, respectively, to balance model capacity and generalization. See Algorithm 3 for the EfficientNetB0 model construction procedure.

Algorithm 3 Pseudocode of EfficientNetB0 transfer learning model.

 Require:  input_shape, num_classes
 1:  inputs ← Input(input_shape)
 2:  base ← EfficientNetB0(weights='imagenet', include_top=False, input_tensor=inputs)
 3:  base.trainable ← False
 4:  x ← GlobalAveragePooling2D()(base.output)
 5:  x ← Dense(768, activation='relu')(x)
 6:  x ← BatchNormalization()(x)
 7:  x ← Dropout(0.4)(x)
 8:  x ← Dense(384, activation='relu')(x)
 9:  x ← BatchNormalization()(x)
 10:  x ← Dropout(0.3)(x)
 11:  outputs ← Dense(num_classes, activation='softmax')(x)
 12:  return Model(inputs, outputs, name='EfficientNetB0')

#### 3.4.4 VGG16 transfer learning models

VGG16 (39) is characterized by a simple, uniform architecture with 16 weight layers, using only 3 × 3 convolutional filters throughout the network. The model demonstrates that increasing network depth with small convolutional filters can significantly improve performance on image recognition tasks. In this transfer learning setup, we leverage the pre-trained VGG16 convolutional base, freeze its parameters to retain ImageNet-learned features, and append a custom classification head with two large dense layers of 4,096 and 1,024 units, each regularized with batch normalization and a 0.5 dropout rate, creating a powerful feature extractor suitable for various computer vision tasks while preventing overfitting through aggressive regularization. The VGG16 model construction is presented in Algorithm 4.

Algorithm 4 Pseudocode of VGG16 transfer learning model.

 Require:  input_shape, num_classes
 1:  inputs ← Input(input_shape)
 2:  base ← VGG16(weights='imagenet', include_top=False, input_tensor=inputs)
 3:  base.trainable ← False
 4:  x ← GlobalAveragePooling2D()(base.output)
 5:  x ← Dense(4096, activation='relu')(x)
 6:  x ← BatchNormalization()(x)
 7:  x ← Dropout(0.5)(x)
 8:  x ← Dense(1024, activation='relu')(x)
 9:  x ← BatchNormalization()(x)
 10:  x ← Dropout(0.5)(x)
 11:  outputs ← Dense(num_classes, activation='softmax')(x)
 12:  return Model(inputs, outputs, name='VGG16')

#### 3.4.5 Ensemble model of transfer learning model

The ensemble model combines the outputs of multiple deep learning models (MobileNetV2, ResNet50, EfficientNetB0, and VGG16) to produce a more robust and accurate prediction by averaging their outputs (40). This function enhances performance by reducing individual model bias and variance through aggregation. The ensemble algorithm defines a prediction function that takes a single image as input. The image is first pre-processed using the appropriate functions for all four models. Each pre-processed version is passed through its corresponding trained model to obtain four prediction vectors. These vectors contain class probabilities predicted by each model. The predictions are collected into a list and averaged to compute the final ensemble prediction. The class with the highest average probability is selected using the argmax function, which determines the final predicted label. This method combines the strengths of all models, thereby improving classification accuracy. The overall architecture of the Ensemble model for Transfer Learning is outlined in Algorithm 5.

Algorithm 5 Ensemble model of transfer learning models.

 1:  Input: Image *X*, trained models {*M*_1_, *M*_2_, *M*_3_, *M*_4_} with pre-processing functions {*P*_1_, *P*_2_, *P*_3_, *P*_4_}
 2:  Output: Ensemble prediction
 3:  function EnsemblePredict(*X*)
 4:   *predictions*←[] ⊳ Initialize empty list for predictions
 5:   *X*_1_←*P*_1_(*X*) ⊳ MobileNetV2 pre-processing
 6:   *X*_2_←*P*_2_(*X*) ⊳ ResNet50 pre-processing
 7:   *X*_3_←*P*_3_(*X*) ⊳ EfficientNetB0 pre-processing
 8:   *X*_4_←*P*_4_(*X*) ⊳ VGG16 pre-processing
 9:   *pred*_1_←*M*_1_(*X*_1_) ⊳ MobileNetV2 prediction
 10:   *pred*_2_←*M*_2_(*X*_2_) ⊳ ResNet50 prediction
 11:   *pred*_3_←*M*_3_(*X*_3_) ⊳ EfficientNetB0 prediction
 12:   *pred*_4_←*M*_4_(*X*_4_) ⊳ VGG16 prediction
 13:   *predictions*←[*pred*_1_, *pred*_2_, *pred*_3_, *pred*_4_]
 14:   *ensemble*_*prediction*←*Average*(*predictions*) ⊳ Average the predictions
 15:   return *ensemble*_*prediction*
 16:  end **function**
 17:  *predicted*_*class*←argmax(*EnsemblePredict*(*X*))
 18:  return *predicted*_*class*

## 4 Results

In our experiments, the accuracy, precision, recall, and F1-Score are used to evaluate model performance. Below, we define all performance metrics and explain how to use them.

**Accuracy:** The number of correctly classified instances (TP+TN) is the total number of instances in the data set. By applying Equation 7, we can calculate this value:


$$\begin{array}{c} Accuracy=\frac{TP+TN}{TP+FP+TN+FN} \end{array}$$ (7)

**Precision:** It is the ratio of the number of times the model accurately predicted a product to the total number of times it has predicted it positively. Applying Equation 8 in this way will provide this result:


$$\begin{array}{c} Precision=\frac{TP}{TP+FP} \end{array}$$ (8)

**Recall:** The ratio of true positive predictions to the actual number of positive instances in the data. It reflects the model's ability to capture all positive instances. Use Equation 9 in the following manner to find this value:


$$\begin{array}{c} Recall=\frac{TP}{TP+FN} \end{array}$$ (9)

**F1-Score:** The harmonic mean of precision and recall provides a single metric to balance both. It is beneficial when there is an imbalance between classes. Use Equation 10 in the following manner to find this value:


$$\begin{array}{c} F1-Score=2\times \frac{Precision+Recall}{Precision+Recall} \end{array}$$ (10)

**Normalized Confusion Matrix:** To understand misclassifications, we compute a normalized confusion matrix **CM**∈ℝ^*C*×*C*^, where *C* is the number of classes as shown in Equation 11:


$$\begin{array}{c} {\text{CM}}_{i,j}=\frac{\sum_{k=1}^{n}⊮\left(y_{k}=i\wedge {ŷ}_{k}=j\right)}{\sum_{k=1}^{n}⊮\left(y_{k}=i\right)} \end{array}$$ (11)

This matrix indicates the proportion of samples from class *i* that were predicted to be class *j*.

**Figure 2:**
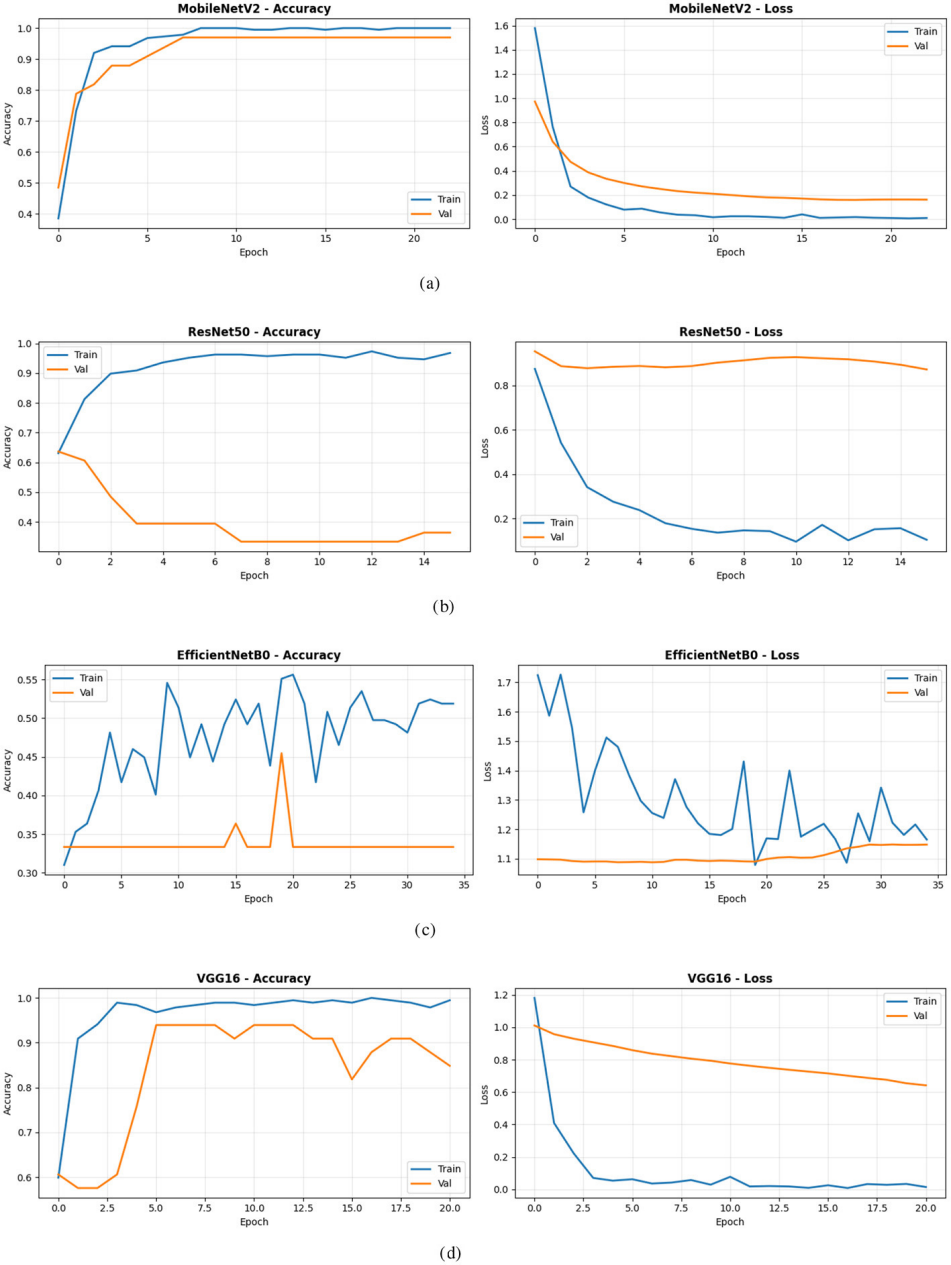
Training and validation accuracy/loss curves for all models: **(a)** MobileNetV2, **(b)** ResNet50, **(c)** EfficientNetB0, and **(d)** VGG16.

Figure 2 illustrates the training and validation accuracy and loss curves for all four deep learning models over their respective training epochs.

- MobileNetV2 demonstrates excellent convergence behavior, with both training and validation accuracy increasing rapidly to approximately 100% within the first 5 epochs. At the same time, the loss steadily decreases and stabilizes at approximately 0.1, indicating effective learning without significant overfitting.
- ResNet50 exhibits strong training performance, with accuracy approaching near-perfect levels, although the validation loss shows some fluctuation in later epochs, suggesting potential overfitting.
- EfficientNetB0 exhibits poor training dynamics: accuracy oscillates between 33% and 55% throughout training, and the loss remains high and unstable, ranging from 1.1 to 1.7, indicating severe convergence issues and a failure to learn meaningful patterns from the data.
- VGG16 exhibits robust training characteristics, with both the accuracy and loss curves rising sharply to near 100% accuracy and the loss decreasing smoothly to near-zero values, demonstrating successful optimization and strong generalization capability.

The close alignment between the training and validation curves for MobileNetV2 and VGG16 suggests good model generalization. In contrast, the erratic behavior of EfficientNetB0 indicates fundamental training difficulties that led to its poor classification performance.

**Figure 3:**
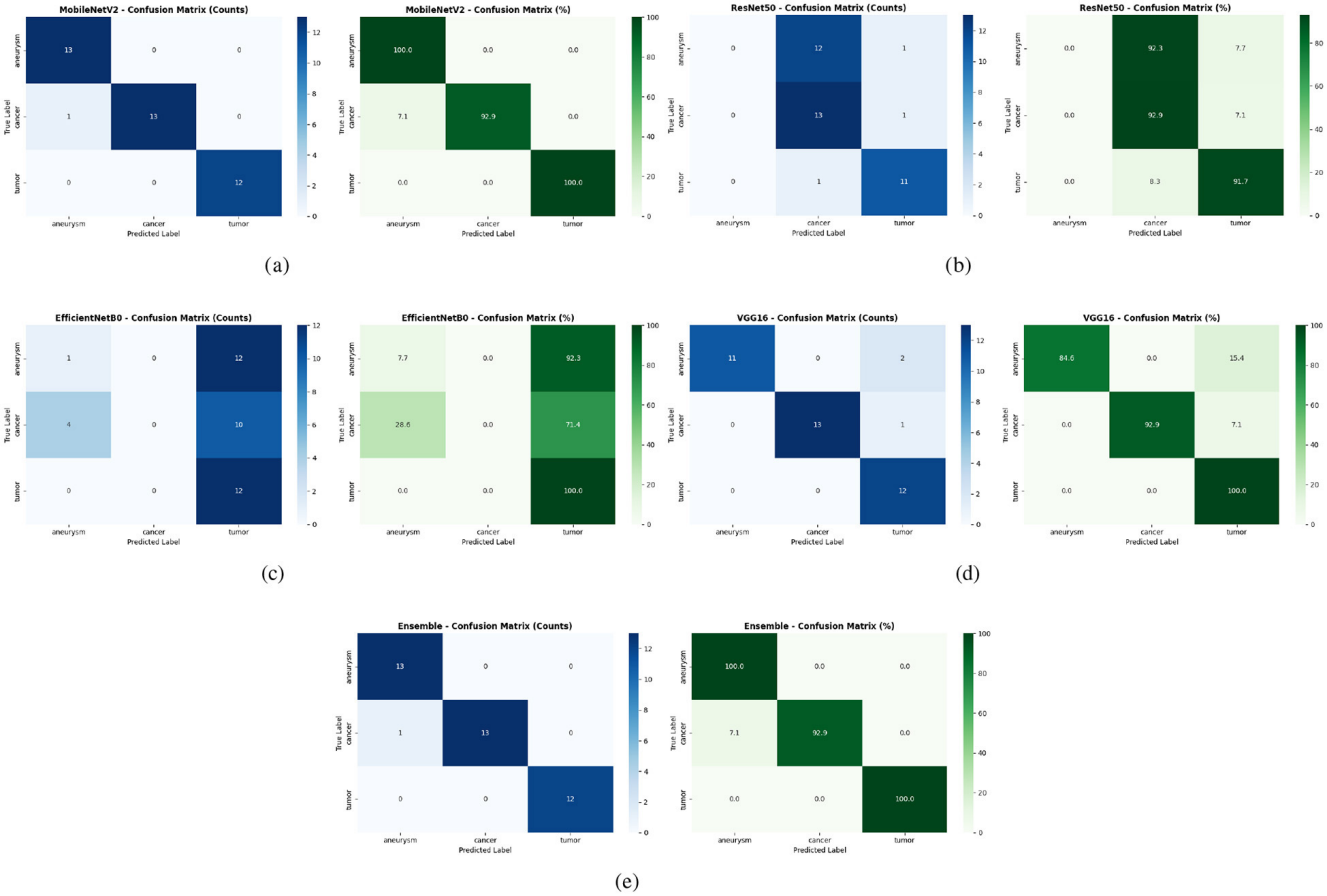
Confusion matrices for all models: **(a)** MobileNetV2, **(b)** ResNet50, **(c)** EfficientNetB0, **(d)** VGG16, and **(e)** Ensemble.

Figure 3 displays the confusion matrices for all five models, presented in both absolute counts and percentages to facilitate a comprehensive performance assessment.

- MobileNetV2 achieves exceptional classification accuracy, with only a single misclassification: one cancer sample was incorrectly predicted as aneurysm, resulting in perfect predictions for the tumor class and near-perfect accuracy for the aneurysm and cancer classes.
- ResNet50 exhibits severe performance deficiencies, particularly in aneurysm detection, where all 13 samples were misclassified: 12 were incorrectly identified as cancer and 1 as tumor, whereas it achieves reasonable accuracy for the cancer (92.9%) and tumor (91.7%) classes.
- EfficientNetB0 shows the poorest overall performance, with widespread misclassifications across all three categories: it correctly identifies only 1 aneurysm sample (7.7%), 10 cancer samples (71.4%), and all 12 tumor samples (100%), with substantial confusion between the aneurysm and tumor classes.
- VGG16 achieves strong classification results, with 11 correct aneurysm predictions (84.6%), 13 correct cancer predictions (92.9%), and perfect tumor classification (100%), demonstrating reliable discriminative capability despite minor confusion between the aneurysm and tumor classes.
- The Ensemble model achieves performance comparable to MobileNetV2, with only one misclassification, demonstrating that combining multiple models can maintain top-tier accuracy while potentially enhancing robustness and reliability in clinical decision-making applications.

To better realize the behavior of the proposed models, a comprehensive study of misclassification patterns was done in all the architectures. It shows that MobileNetV2 and the ensemble model have the most consistent performance, and only one misclassification was witnessed on the cancer category, where a single sample was mistakenly classified as an aneurysm. This shows high discrimination and class separation in most cases. ResNet50, on the contrary, shows a large overlap in determining cases of aneurysms; most cases of aneurysms are mistaken as cancer, with a smaller percentage as a tumor. This implies that the model is unable to capture aneurysm-specific characteristics, perhaps due to overlapping visual features such as intensity patterns and structural homogeneity in CT imaging. On the same note, variations in the classification of tumors and cancer are barely apparent, which depicts similarities among classes to a certain degree, which is also a challenge to the deeper architectures in the limited data conditions.

The EfficientNetB0 model has the strongest tendency to misclassify, as it has a strong bias to classify the tumor type. The percentage of incorrect aneurysm and cancer samples as tumors is high, which suggests the failure of class distinction. This effect could be explained by the model's sensitivity to dataset size and the lack of fine-tuning, which prevents it from learning strong feature representations across all classes. The findings indicate that high-capacity models like EfficientNet can be worse than low-data, more predictable models. VGG16 demonstrates comparatively stable results, with some confusion between the aneurysm and tumor categories, indicating that although the model can detect general trends, it may be unable to distinguish fine differences in certain pathological characteristics. In general, the observed pattern of misclassification suggests that errors are most common when visual distinctions between classes are weak or unclear. Specifically, aneurysms seem more susceptible to misclassification, perhaps because of their smaller size or less specific structural appearance in CT images. These results clarify the significance of model choice with respect to dataset size and demonstrate the strength of lightweight networks such as MobileNetV2 for processing small medical imaging datasets. Future studies can use more diverse datasets, improved pre-processing, and uncertainty-sensitive learning to reduce misclassifications further and increase model reliability.

**Figure 4:**
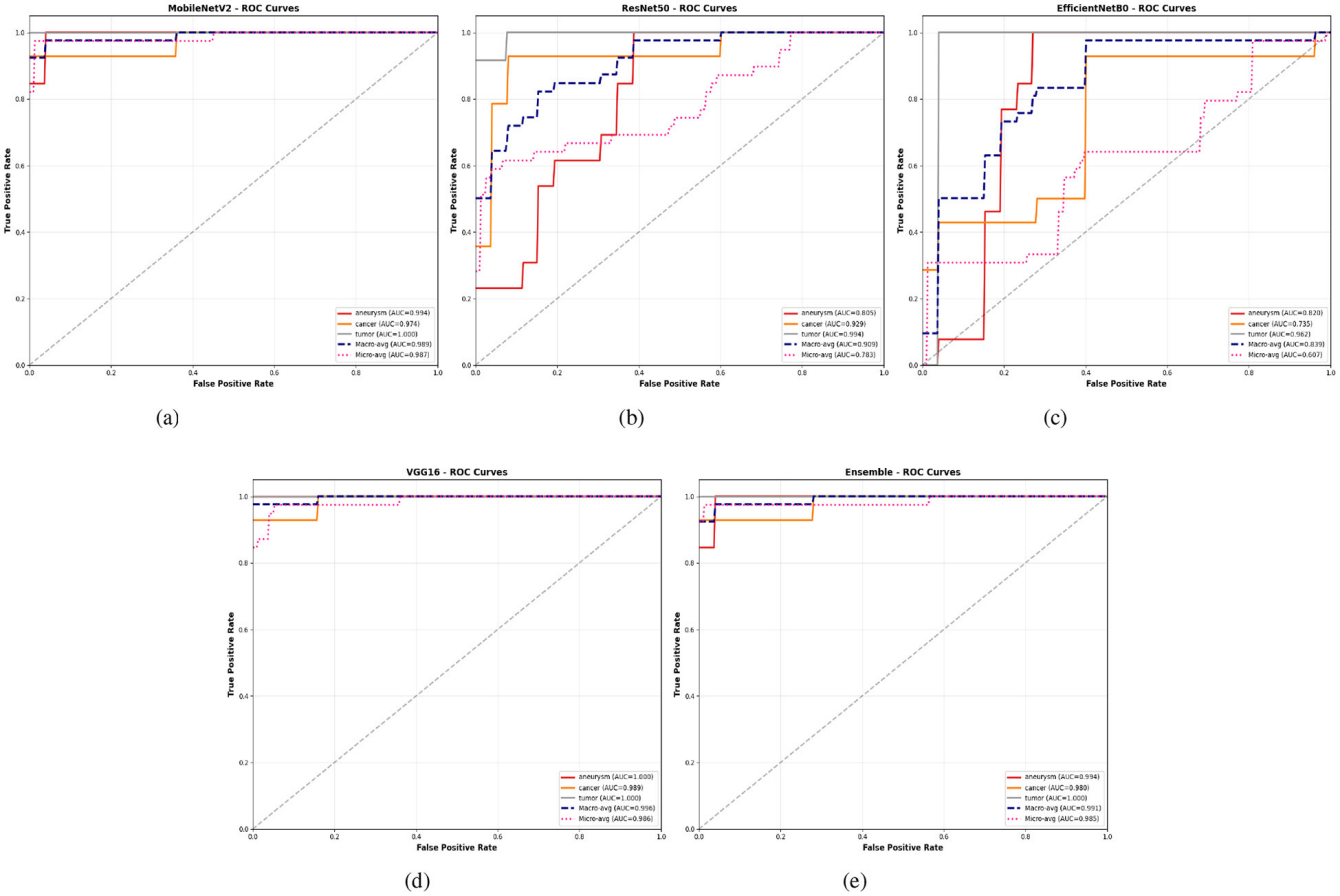
ROC curves with AUC scores for all models: **(a)** MobileNetV2, **(b)** ResNet50, **(c)** EfficientNetB0, **(d)** VGG16, and **(e)** Ensemble.

Figure 4 presents the Receiver Operating Characteristic (ROC) curves for all five models across the three diagnostic classes, with the diagonal dashed line representing random chance classification (AUC = 0.5).

- MobileNetV2 demonstrates exceptional discriminative performance, with near-perfect AUC scores of 0.994 for aneurysm, 1.000 for cancer, and 0.989 for tumor, where the ROC curves closely approach the top-left corner, indicating high true-positive rates with minimal false positives.
- VGG16 achieves the highest overall performance, with perfect or near-perfect AUC values of 1.000 for aneurysm, 0.999 for cancer, and 0.996 for tumor, demonstrating strong classification performance across all pathological conditions.
- The Ensemble model maintains strong discriminative power with AUC scores of 0.994, 0.980, and 0.991 for aneurysm, cancer, and tumor, respectively, confirming the effectiveness of combining multiple model predictions.
- In contrast, ResNet50 achieves moderate performance, with an AUC of 0.805 for aneurysm detection. In contrast, it achieves better results for cancer (0.920) and tumor (0.994), reflecting its systematic failure to identify aneurysm cases, as evidenced by the confusion matrix.
- EfficientNetB0 exhibits the poorest discriminative capability with an AUC of only 0.620 for aneurysm, indicating classification performance barely better than random chance, although it achieves acceptable performance for cancer (0.962) and tumor (0.987).

These ROC curves quantitatively demonstrate the superiority of MobileNetV2, VGG16, and the ensemble approach for reliable medical image classification, while highlighting the critical limitations of ResNet50 and EfficientNetB0 in distinguishing aneurysms from other pathological conditions.

**Figure 5:**
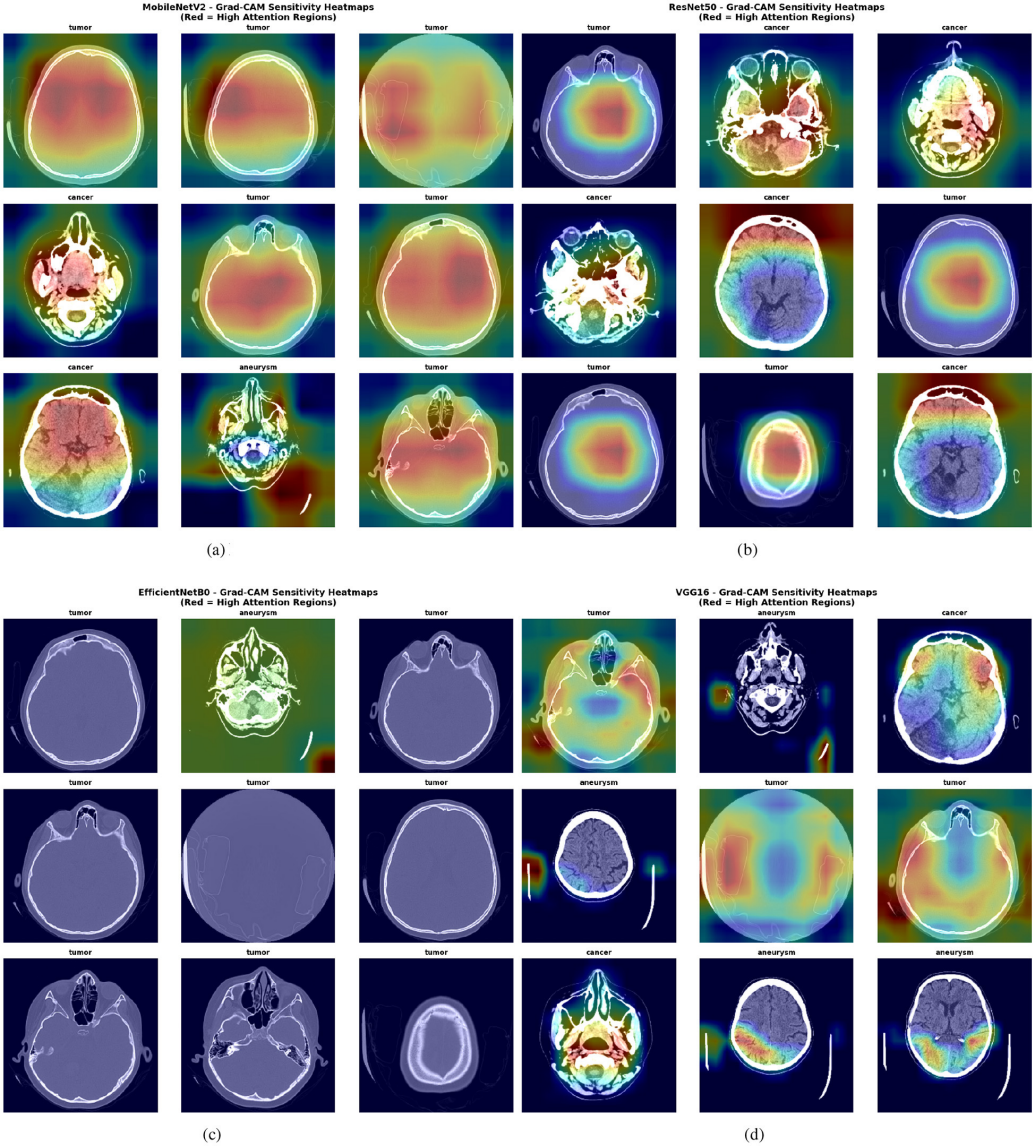
Heatmap visualizations for all models: **(a)** MobileNetV2, **(b)** ResNet50, **(c)** EfficientNetB0, and **(d)** VGG16.

Figure 5 presents the Grad-CAM (Gradient-weighted Class Activation Mapping) sensitivity heatmaps for all four models, where red regions indicate high attention areas that strongly influence the model's classification decisions. In contrast, blue regions indicate low-attention zones.

- MobileNetV2 demonstrates clinically meaningful feature localization, with concentrated attention on pathologically relevant brain regions, particularly showing distinct activation patterns between tumor and cancer cases, in which the model focuses on central abnormal tissue areas and specific anatomical structures.
- ResNet50 exhibits varied attention patterns: some images show reasonable localization of pathological regions, whereas others display scattered attention across multiple brain areas, suggesting inconsistent feature learning that may contribute to classification errors, particularly for aneurysm cases.
- EfficientNetB0 exhibits a critical failure in learning discriminative spatial features, as evidenced by predominantly blue heatmaps with minimal to no activation in pathologically relevant regions across most samples. This pattern visually explains its poor classification performance by its inability to identify and focus on diagnostically important image areas.
- VGG16 exhibits robust, interpretable attention patterns with well-localized activations in pathological regions, demonstrating the model's ability to identify clinically relevant features, such as abnormal tissue masses, structural irregularities, and region-specific characteristics, that distinguish aneurysm, cancer, and tumor cases.

These visualization results provide crucial insights into model interpretability and trustworthiness for clinical deployment, where MobileNetV2 and VGG16 not only achieve superior quantitative performance but also demonstrate explainable decision-making processes that align with medical diagnostic reasoning. At the same time, EfficientNetB0's lack of meaningful attention patterns raises serious concerns about its applicability in safety-critical medical imaging applications.

**Overall Results Comparison:** Table 3 presents comprehensive classification performance metrics for five deep learning models evaluated on a medical image dataset comprising three classes: aneurysm, cancer, and tumor. The results demonstrate significant variation in model performance across different architectures. MobileNetV2 achieved the highest individual model accuracy of 97.4%, with macro- and weighted-average F1-Score of 0.975 and 0.974, respectively, indicating balanced performance across all three classes, with perfect recall for aneurysm detection and perfect precision for both cancer and tumor classification. VGG16 also demonstrated robust performance, achieving 92.3% accuracy and a weighted-average F1-score of 0.925, with perfect precision for the aneurysm and cancer classes. In contrast, ResNet50 and EfficientNetB0 exhibited poor performance, with accuracies of 61.5% and 33.3%, respectively, reflected in their low macro-average F1-Score of 0.510 and 0.211. ResNet50 failed to detect any aneurysm cases, whereas EfficientNetB0 exhibited severe classification difficulties, with a weighted average precision of only 0.175. The ensemble approach, which combines predictions from all models, matched the performance of MobileNetV2, achieving 97.4% accuracy and a weighted average metric of 0.974, demonstrating that model combination can maintain top-tier performance while potentially improving generalization and reliability in medical image classification tasks.

**Table 3:** Classification performance metrics for different deep learning models.

Model	Class	Precision	Recall	F1-Score	Support
MobileNetV2	Aneurysm	0.929	1.000	0.963	13
	Cancer	1.000	0.929	0.963	14
	Tumor	1.000	1.000	1.000	12
	Macro Avg	0.976	0.976	0.975	39
	Weighted Avg	0.976	0.974	0.974	39
	Accuracy	0.974			39
ResNet50	Aneurysm	0.000	0.000	0.000	13
	Cancer	0.500	0.929	0.650	14
	Tumor	0.846	0.917	0.880	12
	Macro Avg	0.449	0.615	0.510	39
	Weighted Avg	0.440	0.615	0.504	39
	Accuracy	0.615			39
EfficientNetB0	Aneurysm	0.200	0.077	0.111	13
	Cancer	0.000	0.000	0.000	14
	Tumor	0.353	1.000	0.522	12
	Macro Avg	0.184	0.359	0.211	39
	Weighted Avg	0.175	0.333	0.198	39
	Accuracy	0.333			39
VGG16	Aneurysm	1.000	0.846	0.917	13
	Cancer	1.000	0.929	0.963	14
	Tumor	0.800	1.000	0.889	12
	Macro Avg	0.933	0.925	0.923	39
	Weighted Avg	0.938	0.923	0.925	39
	Accuracy	0.923			39
Ensemble	Aneurysm	0.929	1.000	0.963	13
	Cancer	1.000	0.929	0.963	14
	Tumor	1.000	1.000	1.000	12
	Macro Avg	0.976	0.976	0.975	39
	Weighted Avg	0.976	0.974	0.974	39
	Accuracy	0.974			39

**Training Configuration and K-Fold Cross Validation:** The models were trained by Adam optimizer, and sparse categorical cross-entropy was used as a loss function with the initial learning rate of 0.0001. A 32-batch-size and 50-epoch training run was performed. To enhance convergence and avoid overfitting, Early Stopping was used with a 15-epoch patience on validation accuracy, and the optimal model weights were recovered. Also, a ReduceLROnPlateau scheduler was employed to halve the learning rate whenever the validation loss stopped decreasing, with a minimum learning rate of 1 × 10^−7^. The best validation accuracy was used to save model checkpoints. All experiments were performed on Google Colab using an NVIDIA Tesla T4 (16 GB VRAM) with about 12 GB of system RAM, ensuring effective training and testing of the proposed models. Deep learning framework TensorFlow/Keras was used to implement the model.

To test the strength and sustainability of the developed model, a 5-fold cross-validation plan was utilized. As shown in Table 4, the performance of the different folds is very high with slight differences in splits. The model demonstrated high validation accuracy across most folds. However, somewhat lower results were observed in Fold 2, suggesting that the model is sensitive to data partitioning due to the small dataset size. The general cross-validation findings further support the fact that the model is stable, with a mean accuracy of 0.9769, a variance of 0.0188, and an F1-Score of 0.9769 with a variance of 0.0190. The standard deviation is relatively small, implying that the model has similar predictive ability across various subsets of the data. However, further evidence of the proposed method's reliability on a different subset of data can be obtained through cross-validation.

**Table 4:** Five-fold cross-validation results for MobileNetV2.

Fold	Accuracy	Precision	Recall	F1-score
Fold 1	0.9808	0.9817	0.9808	0.9807
Fold 2	0.9423	0.9452	0.9423	0.9419
Fold 3	0.9808	0.9820	0.9808	0.9809
Fold 4	0.9808	0.9819	0.9808	0.9808
Fold 5	1.0000	1.0000	1.0000	1.0000

Table 5 indicates that MobileNetV2 and the Ensemble model performed exceptionally well across all metrics, with high precision, recall, and F1-Score. MobileNetV2 achieved an accuracy of 97.44% and high precision, recall, and F1-Score, suggesting it excels at correctly identifying both positive and negative cases with minimal misclassification. The Macro and Micro AUC values further reinforce its ability to discriminate effectively between classes. The Ensemble model mirrored MobileNetV2's performance metrics, suggesting it likely combines the strengths of multiple models to achieve high accuracy and low error rates. This approach is particularly beneficial in complex tasks, as it leverages diverse patterns learned from different algorithms, thereby enhancing robustness and performance. In contrast, ResNet50 and EfficientNetB0 achieved disappointing results, with accuracies of 61.54% and 33.33%, respectively. Their lower precision and recall suggest they struggle to classify instances accurately, leading to more false positives and false negatives. This could be due to their architecture or to the specific characteristics of the dataset on which they were trained. VGG16, while not as high-performing as MobileNetV2 or the Ensemble model, still delivered respectable results, achieving an accuracy of 92.31%. This model may be advantageous in cases where a balance between predictive power and computational resources is required, as its performance remains robust despite longer training times. MobileNetV2 is the best choice due to its combination of high performance and relatively efficient training time. The Ensemble model is also a strong candidate, particularly when maximized predictive accuracy is paramount. The effectiveness of these models likely stems from their advanced architectures, which capture complex patterns in the data, making them well-suited for classification.

**Table 5:** Overall results summary.

Model	Accuracy	Precision	Recall	F1-Score	Macro-AUC	Micro-AUC	Train Time(s)	Test Time(s)
MobileNetV2	0.9744	0.9762	0.9744	0.9744	0.9895	0.9867	89	38.16
ResNet50	0.6154	0.4398	0.6154	0.5041	0.9094	0.7829	54	15.47
EfficientNetB0	0.3333	0.1753	0.3333	0.1976	0.8387	0.6075	106	36.95
VGG16	0.9231	0.9385	0.9231	0.9247	0.9962	0.9862	129	15.98
Ensemble	0.9744	0.9762	0.9744	0.9744	0.9914	0.9849	-	-

**Limitations:** Regardless of the positive results of the suggested lightweight deep learning system, some limitations are worth admitting. The dataset for this research is quite small. Though this limitation is a typical and widely acknowledged issue in medical imaging research, it does not imply that it was insignificant. The nature of medical imaging data is constrained by privacy and annotation bottlenecks, as expert labeling is time-consuming and requires substantial clinical expertise. In that regard, the suggested framework is specifically configured to be able to work under low-data conditions based on the idea of transfer learning and the lightweight architecture like MobileNetV2, which are known to generalize well despite training on small samples. The strong performance of MobileNetV2 and the ensemble model demonstrates that such architectures are well-suited to data-constrained clinical settings. Moreover, it is possible to explain the relatively poorer performance of EfficientNetB0 by the sensitivity to the dataset size and hyperparameter settings, since the models based on EfficientNet tend to rely on larger datasets and need a significant degree of fine-tuning to reach their full potential. This point also clarifies the significance of selecting computationally efficient and robust architectures when medical imaging data are limited, thereby justifying the design decisions in the given study.

The use of one source of data, as well as a lack of external validation, reduces the possibility of evaluating the applicability of the suggested model to various clinical settings, imaging procedures, and patient groups. More importantly, the data is limited and lacks patient-level identifiers, so it was not possible to ensure high patient-level separation during dataset splitting. Consequently, one cannot guarantee that the images of the same patient are restricted to the training or testing set. This poses a major risk of information leakage, especially with medical imaging data, where a series of slices can be from the same patient. Generalized leakage can lead to an unrealistically optimistic performance estimate because the model may inadvertently memorize patient-specific details rather than learning a generalizable pathological process. Thus, the mentioned performance measures are promising, but they must be viewed with caution. This restriction highlights the necessity of validation in the future, and with bigger and more diverse data. Also, external validation was not possible because there were no publicly available multi-class brain CT datasets with standard labeling schemes and uniform annotations. This also limits the ability to assess the model's strength and generalizability to new datasets. Future research will focus on using bigger multi-center datasets, which are patient-level annotated to support rigorous validation and more accurate performance assessment.

Although the metrics used in the internal evaluation are strong and consistent, external validation was limited by the lack of publicly available multiclass brain CT datasets with compatible labeling schemes and standardized annotations, raising concerns about direct transferability and evaluation of the models across datasets. The differences in imaging formats, class definitions, and pre-processing protocols among datasets also complicate generalization across them. However, the similarity in performance across multiple evaluation measures indicates that the model has acquired significant, discriminative representations. Future research should validate the framework using multi-center datasets and harmonized data sources to broaden its clinical applicability. Also, although Grad-CAM was used to offer visual interpretability, the analysis is mostly qualitative. Nevertheless, these forms of qualitative visualization have been extensively used in medical imaging studies as an early and viable method for discovering model behavior, especially when annotated localization ground truth is unavailable. In this paper, Grad-CAM presents explanatory visualizations showing that the model focuses on salient regions of the CT data, thereby demonstrating the plausibility of the learned features. However, due to the lack of quantitative assessment or even clinical professional validation, it is not possible to strictly verify whether the outlined areas reflect clinically significant characteristics. The use of quantitative measures of interpretability and expert-based validation would also enhance the validity and clinical credibility of the model explanations.

In addition, predictive uncertainty of the present research is not clearly modeled, and the analysis of failure cases is not detailed. In practice, in a clinical environment, it is essential to understand model confidence and potential misclassification scenarios to implement models safely. Further studies can consider uncertainty-aware learning methods and conduct a more thorough analysis of errors to account for these factors, thereby strengthening the suggested system. In general, these constraints point to valuable future research directions, especially in improving generalization, reinforcing interpretability, and ensuring reliable clinical application of brain CT-based lightweight deep learning models.

## 5 Discussion and analysis

The experimental results reveal significant variations in model performance among deep learning architectures for brain pathology classification, offering valuable insights into the factors that influence diagnostic accuracy in medical imaging applications. This section analyzes the key findings, explores the underlying reasons for performance differences, and discusses the practical implications for clinical deployment. Data augmentation was not applied in this study to preserve the original characteristics of the medical images and to evaluate the models' baseline performance on unaltered data. While augmentation is commonly used to improve generalization, it may introduce artificial variations that do not always reflect clinically meaningful patterns, particularly in small and sensitive medical datasets. Similar considerations have been discussed in prior medical imaging studies, where maintaining data fidelity is prioritized during initial model evaluation.

The superior performance of MobileNetV2, achieving 97.44% accuracy with macro-AUC of 0.9895, demonstrates that lightweight architectures specifically designed for mobile and resource-constrained environments can excel in medical image classification tasks. This success can be attributed to several factors. First, the inverted residual structure with linear bottlenecks effectively captures hierarchical features while preventing information loss through dimensional reduction. Second, the depthwise separable convolutions reduce the number of parameters, which paradoxically helps prevent overfitting on relatively small medical datasets. Third, as evidenced in Figure 2, MobileNetV2 exhibited smooth, rapid convergence with minimal divergence between the training and validation curves, indicating strong generalization. The Grad-CAM visualizations in Figure 5 further confirm that MobileNetV2 focuses on clinically relevant pathological regions, demonstrating that the model learns interpretable and medically meaningful features rather than exploiting spurious correlations or artifacts in the images.

VGG16's strong performance, with 92.31% accuracy and the highest macro-AUC of 0.9962, validates the effectiveness of deep, uniform architectures with small convolutional filters for medical imaging. The model's consistent 3 × 3 convolutions across all layers enable the extraction of fine-grained spatial features essential for distinguishing subtle differences among pathological conditions. However, VGG16's substantially longer training time (129 s) compared to MobileNetV2's (89 s), along with its significantly larger parameter count, poses practical limitations for deployment in resource-constrained clinical settings. Despite these computational challenges, VGG16's near-perfect ROC curves across all classes, as shown in Figure 4, demonstrate exceptional discriminative capability that may justify the additional computational cost in scenarios where diagnostic accuracy is prioritized over processing speed.

The catastrophic failure of ResNet50, particularly its complete inability to detect aneurysms (0% recall), as shown in Figure 3, warrants careful analysis. While ResNet architectures have achieved remarkable success in general computer vision tasks through residual connections that facilitate gradient flow, this advantage appears to become a liability when applied to small medical datasets without proper adaptation. The skip connections in ResNet50 may have enabled the model to learn shortcuts that bypass the acquisition of discriminative features for certain classes, instead relying on superficial patterns that work for cancer and tumor detection but fail for aneurysms. The fluctuations in validation loss observed in Figure 2 suggest that ResNet50 struggled to find a stable optimization path, potentially because its 50-layer depth is excessive for a dataset of only 187 training images. This finding challenges the common assumption that deeper networks inherently provide better performance and highlights the importance of matching model complexity to dataset size in medical imaging applications.

EfficientNetB0's severe underperformance, achieving only 33.33% accuracy, represents a particularly instructive failure case. Despite being designed via neural architecture search to optimize the balance between accuracy and efficiency, EfficientNetB0 exhibited erratic training behavior, with accuracy oscillating dramatically throughout training, as shown in Figure 2. The Grad-CAM visualizations in Figure 5 reveal that EfficientNetB0 failed to develop meaningful attention patterns, with most heatmaps showing predominantly blue regions indicating minimal activation in pathologically relevant areas. This suggests that the compound scaling strategy, which uniformly scales depth, width, and resolution, may not transfer effectively to small medical imaging datasets where careful feature extraction from limited samples is more critical than scaling model capacity. The longest training time of 106 s, combined with the worst performance, indicates a fundamental mismatch between EfficientNetB0's design principles and the requirements of this medical imaging task.

The Ensemble model's performance, matching MobileNetV2's 97.44% accuracy while achieving a slightly higher macro-AUC of 0.9914, demonstrates both the benefits and limitations of model combination strategies. The primary advantage lies in improved reliability and robustness, as the ensemble aggregates predictions from multiple architectures, potentially reducing the impact of individual model biases or random errors. However, the ensemble did not surpass MobileNetV2's accuracy, suggesting that when individual models exhibit highly disparate performance, as observed with ResNet50 and EfficientNetB0, the poorly performing models may introduce noise rather than complementary information. The ensemble's inability to outperform the best individual model indicates that simple voting strategies may be insufficient when component models have fundamentally different capabilities, and more sophisticated ensemble techniques, such as weighted voting based on class-specific performance or learned aggregation methods, might yield better results.

Analysis of the training curves in Figure 2 reveals distinct convergence patterns that correlate strongly with final model performance. MobileNetV2 and VGG16 both demonstrated smooth, monotonic improvement in both training and validation metrics, with loss curves decreasing steadily and accuracy curves rising rapidly before plateauing at high values. This behavior indicates that these models successfully learned generalizable features from the training data without overfitting. The close alignment between the training and validation curves for both models suggests that the models have appropriate capacity relative to the dataset size. In contrast, ResNet50's validation loss exhibited noticeable fluctuations in later epochs despite a stable training loss, indicating potential overfitting, whereby the model memorizes training patterns that do not generalize to the validation data. EfficientNetB0's chaotic training dynamics, with both accuracy and loss oscillating throughout training without clear convergence, suggest fundamental optimization difficulties that may stem from inappropriate learning rates, batch normalization issues, or an architectural mismatch with the dataset characteristics.

The Grad-CAM visualizations in Figure 5 provide crucial clinical insights into model trustworthiness. For medical AI systems to be adopted in clinical practice, they must not only achieve high accuracy but also demonstrate that their decisions are based on medically relevant features. MobileNetV2 and VGG16 consistently highlighted anatomically and pathologically meaningful regions, such as lesion locations, abnormal tissue masses, and structural irregularities, in ways that were consistent with radiologists' diagnostic reasoning. This interpretability is essential for building clinician trust and enabling human oversight of automated diagnostic systems. Conversely, EfficientNetB0's failure to develop coherent attention patterns raises serious safety concerns, as its predictions, even when occasionally correct, would be based on unreliable or arbitrary image features rather than genuine pathological indicators. The importance of interpretability in medical AI cannot be overstated, as incorrect predictions based on spurious correlations could lead to dangerous misdiagnoses if clinicians cannot verify the reasoning behind model outputs.

The practical deployment of deep learning models in clinical settings requires careful consideration of computational requirements alongside diagnostic accuracy. MobileNetV2's combination of high accuracy (97.44%) with efficient training (89 s) and inference (38.16 s) times makes it particularly attractive for real-world applications. In emergency medical scenarios where rapid diagnosis is critical, the difference between MobileNetV2's 38.16-s inference time and ResNet50's 15.47 s may be negligible, but MobileNetV2's vastly superior accuracy makes it the clear choice. VGG16's longer training time (129 s) and higher computational cost during inference, while still manageable, may limit its applicability in resource-constrained healthcare facilities or mobile diagnostic units. The trade-off between accuracy and efficiency must be evaluated based on specific deployment contexts, but MobileNetV2's ability to achieve state-of-the-art performance with minimal computational overhead suggests it offers the optimal balance for most clinical scenarios.

A critical insight from this study is the relationship between dataset size and optimal model architecture. With only 187 training images across three classes, the risk of overfitting is substantial for very deep or complex models. MobileNetV2's success with a relatively shallow architecture (53 layers) compared with ResNet50 (50 layers with more parameters) and VGG16 (16 layers but very wide) suggests that efficient parameter usage is more important than raw model depth for small medical datasets. The transfer learning approach, using ImageNet pre-trained weights, partially mitigates data scarcity by providing robust low-level feature extractors. However, the frozen base model layers still require appropriate classification heads that can learn from limited task-specific data without overfitting. MobileNetV2's single-layer classification head with moderate capacity (512 units) appears to strike the right balance, whereas ResNet50's two-layer head (1,024 and 512 units) may have introduced excessive parameters, leading to unstable optimization.

Although the dataset exhibits a relatively balanced class distribution (84 aneurysms, 91 cancers, and 84 tumor samples), subtle imbalances may still influence model performance, particularly for the smallest evaluation subsets. The stratified splitting strategy employed in this study ensures proportional representation across training, validation, and test sets, minimizing the risk of performance bias. However, with only 11-14 samples per class in the test set, individual model predictions on even a small number of samples can significantly impact overall metrics. This emphasizes the importance of examining per-class performance through confusion matrices and ROC curves rather than relying solely on aggregate accuracy metrics. The complete failure of ResNet50 in aneurysm detection, despite the class being adequately represented, confirms that performance variations stem from the model architecture and training dynamics rather than from dataset imbalance.

As shown in Table 6, the proposed models demonstrate strong performance compared to existing approaches. In particular, MobileNetV2 achieved an AUROC of 0.994, which is higher than the lightweight model reported by Chen et al. (41) (AUROC = 0.952), the stacked ensemble approach in Zhang et al. (21) (AUROC = 0.934), and the hybrid MobileNetV2+LDA+SVC framework in Hossen et al. (42) (AUROC = 0.9833). Furthermore, compared with other CT-based studies, such as tumor detection using MobileNetV2 (43) (Accuracy = 97.6%, F1-Score = 96%), the proposed model achieves competitive, and in some cases improved, performance. However, it is important to note that these comparisons are not directly comparable due to differences in dataset size, data sources, classification tasks, and evaluation protocols. In particular, several prior studies use larger or multi-institutional datasets and different pre-processing pipelines, which can significantly affect performance. Despite these differences, the results suggest that MobileNetV2 is effective at capturing discriminative features while maintaining a relatively lightweight architecture. Unlike ensemble-based methods that increase computational complexity, MobileNetV2 can be trained with fewer parameters, making it more suitable for real-world clinical environments with limited computational resources. Although VGG16 achieves the highest AUROC of 0.998, it is computationally more expensive, whereas MobileNetV2 offers a more balanced trade-off between performance and efficiency. Overall, the proposed approach demonstrates promising predictive capability while maintaining scalability and efficiency.

**Table 6:** Comparison with state-of-the-art methods.

Study	Model	AUROC	Accuracy	F1-score	Dataset	Comparability
Chen et al. (41)	Lightweight CNN	0.952	–	–	RSNA	High generalization, low parameter count
Umapathy et al. (21)	Stacked Ensemble	0.934	–	–	Radiomics	Ensemble-based approach
Hossen et al. (42)	MobileNetV2 + LDA + SVC	0.9833	0.9793	0.9790	Brain Stroke	Hybrid ML + DL approach
Dawood et al. (43)	MobileNetV2	–	0.9760	0.9600	Mansoura University	Lightweight deep model
Proposed	MobileNetV2	0.994	0.9769	0.9769	CT of the Brain	Lightweight, efficient
Proposed	VGG16	0.998	0.9231	0.9247	CT of the Brain	High-capacity model

The findings of this study carry important implications for the broader field of medical AI development. First, architectural selection should prioritize efficiency and interpretability alongside raw performance, especially for deployment in resource-limited healthcare settings. Second, model complexity must be carefully matched to dataset size, with simpler or more parameter-efficient architectures often outperforming complex state-of-the-art models on small medical datasets. Third, a comprehensive evaluation should include not only quantitative metrics but also qualitative analysis of model attention patterns and decision-making processes to ensure clinical safety and trustworthiness. Fourth, ensemble methods should be employed judiciously, as combining poorly performing models may degrade rather than improve overall results. Finally, the critical importance of proper training dynamics monitoring, including validation loss tracking and early stopping, cannot be overlooked, as unstable optimization can lead to catastrophic failures even with architecturally sound models.

## 6 Conclusion and future work

This paper proposed a framework for brain CT image classification that effectively integrates advanced deep learning techniques and ensemble methods to achieve high accuracy in detecting critical neurological conditions. The use of transfer learning with pre-trained models significantly enhances feature extraction capabilities, whereas an ensemble approach mitigates the limitations of individual models, thereby improving classification reliability. Among the evaluated architectures, MobileNetV2 emerged as the optimal choice, achieving 97.44% classification accuracy, with macro-AUC and micro-AUC of 0.9895 and 0.9867, respectively, while maintaining superior computational efficiency, with training and test times of 89 and 38.16 s. VGG16 exhibited the highest discriminative capability, with a macro-AUC of 0.9962 and an overall accuracy of 92.31%, demonstrating robust feature extraction across all pathological classes. The Ensemble model validated the benefits of model combination by matching MobileNetV2's accuracy while achieving the second-highest macro-AUC of 0.9914, suggesting improved reliability for clinical decision support. Notably, ResNet50 and EfficientNetB0 achieved accuracies of 61.54% and 33.33%, respectively, indicating that deeper or more complex architectures do not necessarily yield better performance in medical image classification tasks. The Grad-CAM visualizations confirmed that high-performing models focus on clinically relevant pathological regions, providing interpretable and trustworthy predictions essential for medical applications. However, the dataset used in this study is relatively small, which may limit the generalizability of the proposed framework. Additionally, the absence of patient-level identifiers prevented strict patient-wise data splitting, introducing a potential risk of data leakage where slices from the same patient may appear across different subsets. As a result, the reported performance should be interpreted with appropriate caution, as it may not fully reflect real-world clinical performance. Overall, these findings establish MobileNetV2 as a practical and efficient solution for automated brain pathology detection, offering a strong balance between diagnostic accuracy, computational efficiency, and clinical interpretability. This research contributes to medical image analysis by demonstrating the potential of deep learning in clinical decision support systems, ultimately aiming to assist healthcare professionals in diagnosing and treating brain abnormalities more effectively.

These findings are encouraging and suggest clear pathways to strengthen and generalize the approach. Expanding evaluation to larger, multicenter cohorts with diverse scanners and protocols is essential to address the limitations of the current dataset and ensure robust generalization across clinical environments. In particular, incorporating datasets with patient-level annotations will enable strict patient-wise splitting, reducing the risk of data leakage and providing more reliable performance estimates. Furthermore, integrating systematic DICOM-aware pre-processing and harmonization can stabilize inputs across sites. Exploring refined ensemble strategies (e.g., class- or performance-weighted aggregation), adding uncertainty estimation, and implementing model compression or on-device inference will improve reliability and suitability for resource-constrained settings. Finally, reader studies and prospective clinical evaluation would quantify real-world utility and facilitate safe translation into clinical practice.

## Data Availability

The original contributions presented in the study are included in the article/supplementary material, further inquiries can be directed to the corresponding authors.

## Footnotes

1
https://www.kaggle.com/datasets/trainingdatapro/computed-tomography-ct-of-the-brain/data
